# Pre-thrombolysis serum sodium concentration is associated with post-thrombolysis symptomatic intracranial hemorrhage in ischemic stroke patients

**DOI:** 10.3389/fneur.2024.1341522

**Published:** 2024-05-31

**Authors:** Xiaolan Wu, Zhuangzhuang Jiang, Dongjuan Xu, Rufang Zhang, Hongfei Li

**Affiliations:** Department of Neurology, Dongyang People’s Hospital, Affiliated to Wenzhou Medical University, Dongyang, China

**Keywords:** serum sodium, symptomatic intracranial hemorrhage, intravenous thrombolysis, ischemic stroke, early infarct signs

## Abstract

**Background and aim:**

Symptomatic intracranial hemorrhage (sICH) was the most serious complication associated with alteplase intravenous thrombolysis (IVT) in acute ischemic stroke (AIS) patients. However, the relationship between serum sodium levels and post-thrombolysis symptomatic intracranial hemorrhage has not been investigated. Therefore, the aim of this study was to investigate the relationship between pre-thrombolysis serum sodium levels and sICH after IVT, as well as to explore the optimal pre-thrombolysis serum sodium levels for lowering the risk of sICH following IVT.

**Methods:**

From July 1, 2017 to April 30, 2023, out-of-hospital AIS patients who received IVT in the emergency department were enrolled in this study. Serum sodium levels were measured at admission prior to IVT, and National Institutes of Health Stroke Scale scores were continuously assessed during and after thrombolysis. Routine follow-up neuroimaging was performed between 22 to 36 h after IVT. Initially, three logistic regression models and restricted cubic splines (RCS) were established to investigate the relationship between serum sodium levels and post-thrombolysis sICH. Furthermore, to evaluate the predictive value of serum sodium for post-thrombolysis sICH, we compared area under the receiver operating characteristic curve (AUROC) and net reclassification improvement (NRI) before and after incorporating serum sodium into traditional models. Finally, subgroup analysis was conducted to explore interactions between serum sodium levels and other variables.

**Results:**

A total of 784 AIS patients who underwent IVT were enrolled, among whom 47 (6.0%) experienced sICH. The median serum sodium concentration for all patients was 139.10 [interquartile ranges (IQR): 137.40–141.00] mmol/L. Patients who developed sICH had lower serum sodium levels than those without sICH [138.20(IQR:136.00–140.20) vs. 139.20(IQR:137.40–141.00), *p* = 0.031]. Logistic regression analysis (model 3) revealed a 14% reduction in the risk of post-thrombolysis sICH for every 1 mmol/L increase in serum sodium levels after adjusting for confounding variables (*p* < 0.001). The risk of post-thrombolysis sICH was minimized within the serum sodium range of 139.1–140.9 mmol/L compared to serum sodium concentration below 137.0 mmol/L [odds ratio (OR) = 0.33, 95% confidence interval (CI): 0.13–0.81] in model3. Furthermore, there was a significant trend of decreasing risk for sICH as serum sodium concentrations increased across the four quartiles (*P* for trend = 0.036). The RCS analysis indicated a statistically significant reduction in the risk of sICH as serum sodium levels increased when the concentration was below 139.1 mmol/L. Incorporating serum sodium into traditional models improved their predictive performance, resulting in higher AUROC and NRI values. Subgroup analysis suggested that early infarct signs (EIS) appeared to moderate the relationship between serum sodium and sICH (*p* < 0.05).

**Conclusion:**

Lower serum sodium levels were identified as independent risk factors for post-thrombolysis sICH. Maintaining pre-thrombolysis serum sodium concentrations above 139.1 mmol/L may help reduce the risk of post-thrombolysis sICH.

## Introduction

Acute ischemic stroke (AIS), resulting from arterial occlusion, stands as the most prevalent stroke subtype globally and ranks among the leading causes of disability and mortality worldwide (1). Extensive evidence has underscored the efficacy of intravenous thrombolysis (IVT) with recombinant tissue plasminogen activator (RTPA) in promoting the functional recovery of AIS patients (2). However, symptomatic intracranial hemorrhage (sICH) associated with IVT poses a significant threat to patient outcomes (3).

Numerous studies have delved into the risk factors linked to intracranial hemorrhage following IVT (4–7). However, the majority of identified predictive variables in these studies are non-modifiable, implying that in cases of patients at high risk of sICH following IVT, treatment options are often limited to discontinuing thrombolysis or reducing rtPA dosage (8). The identification of modifiable variables for post-thrombolysis sICH would significantly expand treatment avenues.

Patients with ischemic stroke are often accompanied with hyponatremia due to the activation of syndrome of inappropriate antidiuretic hormone secretion (SIADH) and cerebral salt wasting (CSW) (9, 10). Recent research has indicated a relationship between hyponatremia and the hemorrhagic transformation (HT) following thrombolysis (11). Nevertheless, the relationship between serum sodium concentration and sICH following thrombolysis in ischemic stroke patients remains uncertain. Given that serum natrium levels can be effectively modulated through medical interventions (12, 13), and sICH exerts a more pronounced effect on the long-term prognosis of ischemic stroke patients receiving thrombolytic therapy compared to other types of HT (14), investigating the relationship between serum sodium levels and sICH is crucial. Therefore, this study aims to explore the correlation between pre-thrombolysis serum sodium levels and sICH following IVT, as well as to investigate the optimal pre-thrombolysis serum sodium levels for reducing the risk of sICH after IVT.

## Materials and methods

### Patients

In this retrospective observational study, we focused on consecutive patients experiencing AIS out-of-hospital who underwent rtPA IVT treatment in the emergency department at Dongyang People’s Hospital from July 1, 2017 to April 30, 2023. The study was ethically approved by the Ethics Committee of Dongyang People’s Hospital (2023-YX-384) and adhered strictly to the principles of the Declaration of Helsinki. To fully protect patient privacy, personal information was concealed during data extraction and analysis. Participants were included based on the following inclusion criteria: (1) being 18 years of age or older, (2) having been diagnosed with AIS by experienced neurologists with no evidence of intracranial hemorrhage on cranial non-contrast computed tomography (NCCT) (15), and (3) undergoing alteplase thrombolysis within 4.5 h from the onset of stroke symptoms, in accordance with the guidelines established by the European Stroke Organization (16). Exclusion criteria consisted of: (1) patients undergoing endovascular treatment subsequent to IVT, (2) patients with incomplete clinical data, (3) patients receiving tenecteplase (TNK) intravenous thrombolysis, and (4) patients with onset-to-thrombolysis time exceeding 4.5 h.

### Data collection

We collected a comprehensive set of baseline characteristics, including demographic information, vascular risk factors, prior medication history, clinical data at admission, laboratory test results at admission, RTPA dosage and baseline neuroimaging before IVT. Hypertension, diabetes mellitus, hyperlipemia, ischemic heart disease (IHD) or atrial fibrillation (AF) were considered present if there was a clear medical history or if the condition was definitively diagnosed at discharge. Cigarette smoking was defined as a history of smoking at least one cigarette per day for 6 months or more (17). Alcohol consumption was defined as consuming 15 g or more alcoholic drinks per day in the previous years (18). A prior history of stroke was defined as a history of transient ischemic attack or ischemic stroke (19). In the majority of cases, RTPA was administered following the standard protocol: a dose of 0.9 mg/kg, not exceeding 90 mg total, infused over 60 min, with an initial bolus of 10% of the dose delivered within the first minute (20). However, a small subset of patients received RTPA based on a different guideline: a dose of 0.6 mg/kg, still capped at 90 mg over 60 min, but with an initial bolus of 15% of the dose within the first minute (21). Early infarct signs (EIS) included alterations in signal intensity of brain parenchyma, structural changes or dense cerebral artery sign on cranial NCCT (22). Both neuroimaging and National Institutes of Health Stroke Scale (NIHSS) scores were assessed independently by two experienced neurologists (23). The agreement between them for EIS was evaluated using the kappa statistic, yielding a value of 0.783. Disagreements were resolved through discussion. All included patients received follow-up cranial NCCT or magnetic resonance imaging (MRI) between 22 and 36 h after intravenous thrombolysis. Additionally, in case of any neurological deterioration, an additional cranial NCCT scan was conducted. Symptomatic intracranial hemorrhage was defined, according to the European Cooperative Acute Stroke Study II (24), as any type of intracranial hemorrhage detected on post-IVT imaging coupled with an increase of NIHSS score by at least 4 points compared to baseline or leading to death. To validate previous studies on the relationship between hyponatremia and post-thrombolysis HT in ischemic stroke patients, patients with HT were also included in this study. HT was determined according to the European-Australasian Acute Stroke Study (ECASS) II classification criteria (24).

### Statistical analysis

All continuous variables were represented as medians with interquartile ranges (IQRs) due to non-normal distribution based on a one-sample Kolmogorov–Smirnov test, while categorical variables were expressed as counts and percentages. Baseline characteristics were stratified according to the presence or absence of sICH. Mann–Whitney U-tests were used for continuous variables to assess intergroup differences, while Pearson chi-square test was employed for categorical variables. To explore a potential independent association between sICH and serum sodium levels, three logistic regression models were employed. Based on the intergroup comparison results (variables with *p* < 0.05) and prior literatures (4–7), we have chosen the candidate adjusting variables. Patients were divided into four quartiles (Q1–Q4) according to serum sodium levels (Q1 < 137.4 mmol/L; Q2 137.4–139.0 mmol/L; Q3 139.1–140.9 mmol/L; Q4 ≥ 140.1 mmol/L). Serum sodium concentration was included as both a categorical and continuous variable in this study. Model 1 examined the unadjusted relationship between serum sodium and sICH. Model 2 included additional adjustments for age, sex and AF. Model 3 expanded on the adjustments made in Model 2 to include NIHSS score, early infarct signs, diastolic blood pressure, blood urea nitrogen, and fibrinogen. Restricted cubic splines (RCS) analysis was performed to explore potential linear correlations and dose–response relationships between serum sodium levels and sICH. The area under the receiver operating characteristic curve (AUROC) and continuous net reclassification improvement (NRI) were calculated to evaluate whether serum sodium enhances the predictive performance of traditional post-thrombolysis sICH prediction models. Interaction analyses were conducted separately to explore potential moderating variables in the relationship between serum sodium and sICH, integrating multiplicative interaction terms and likelihood-ratio tests to assess interaction presence. Statistical significance was set at a two-tailed *p*-value less than 0.05. All analyses were performed using SPSS version 26.0 and R software 4.1.1.

## Results

### Baseline patient characteristics

**Figure 1:**
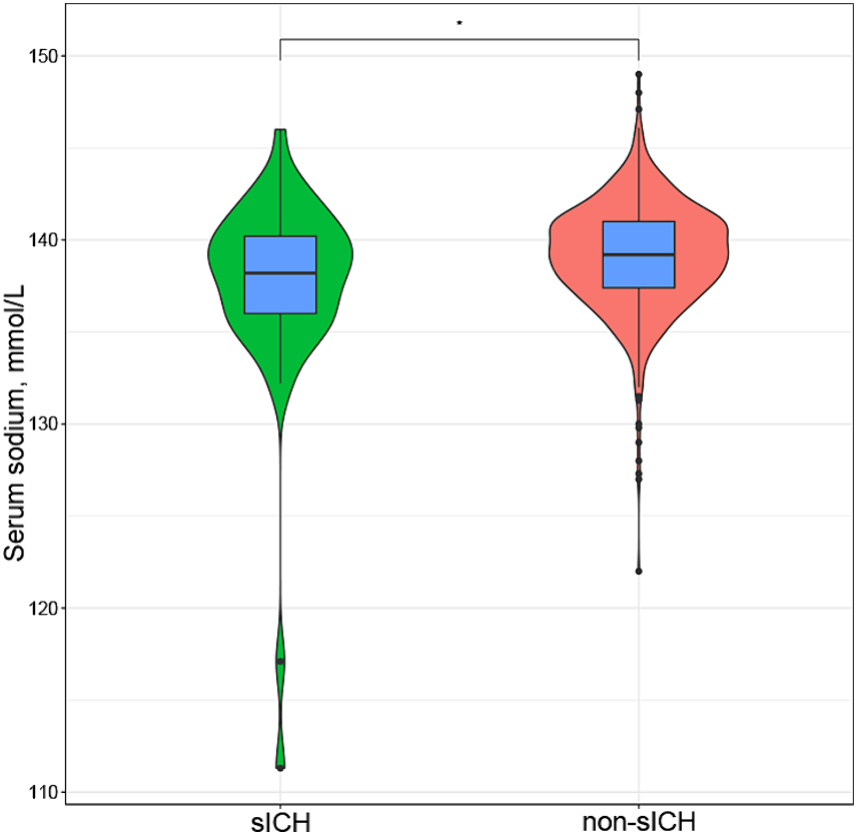
Box plot and violin plot shown difference between serum sodium levels in sICH group and non-sICH group after thrombolysis in ischemic stroke patients [138.20(IQR:136.00–140.20) vs. 139.20(IQR:137.40–141.00), *p* = 0.031]. sICH, symptomatic intracranial hemorrhage.

A total of 888 patients with AIS who underwent IVT treatment were initially considered for this study. Among them, 36 patients had missing data (baseline laboratory test results were missing for 26 patients, baseline neuroimaging before thrombolysis were missing for 8 patients, post-thrombolysis neuroimaging and NIHSS scores were missing for 2 patients), and 68 patients underwent additional endovascular treatment following IVT. Finally, the study included 784 patients and no statistically significant differences were found in the comparison between excluded and included cases. The baseline characteristics of included patients are shown in Table 1. Of these participants, 502 (64.0%) were male, with an average age of 69.50 years (IQR: 59.00–78.00). Forty-seven individuals (6.0%) experienced sICH. The median serum sodium concentration across all 784 patients was 139.10 mmol/L (IQR: 137.40–141.00). Serum sodium levels were significantly lower in individuals with sICH compared to those without sICH without any adjustment for other factors [138.20(IQR:136.00–140.20) vs. 139.20(IQR:137.40–141.00), *p* = 0.031], as vividly depicted in Figure 1. Participants with sICH demonstrated elevated baseline diastolic blood pressure (DBP), fibrinogen, blood urea nitrogen (BUN) and NIHSS scores, alongside a heightened probability of advanced age, the presence of AF, and the manifestation of EIS (*p* < 0.05). A total of 149 individuals (19%) experienced post-thrombolysis HT. It was observed that serum sodium levels were significantly lower in individuals with HT compared to those without HT [138.50(IQR:136.80, 140.20) vs. 139.50(IQR:137.70, 141.00), *p* = 0.004] (Supplementary Table S1). Serum sodium concentration remained associated with the occurrence of HT after adjusting for multiple potential confounders (*p* < 0.05) (Supplementary Table S2).

**Table 1:** Baseline patient characteristics categorized by symptomatic intracranial hemorrhage.

Variables	Total (*N* = 784)	sICH (*N* = 47)	Non-sICH (*N* = 737)	*p*
Demographic variables				
Age (years), median (IQR)	69.50 (59.00,78.00)	77.00 (65.50, 84.50)	69.00 (59.00, 78.00)	<0.001
Female, *n* (%)	282 (36.0%)	16 (34.0%)	266 (36.1%)	0.776
Vascular risk factors				
Smoking, *n* (%)	140 (17.9%)	9 (19.1%)	131 (17.8%)	0.811
Drinking, *n* (%)	165 (21.0%)	12 (25.5%)	153 (20.8%)	0.436
Hypertension, *n* (%)	537 (68.5%)	29 (61.7%)	508 (68.9%)	0.301
Diabetes mellitus, *n* (%)	141 (18.0%)	10 (21.3%)	131 (17.8%)	0.544
Hyperlipidemia, *n* (%)	272 (34.7%)	17 (36.2%)	255 (34.6%)	0.826
Ischemic heart disease, *n* (%)	116 (14.8%)	11 (23.4%)	105 (14.2%)	0.086
Atrial fibrillation, *n* (%)	130 (16.6%)	19 (40.4%)	111 (15.1%)	<0.001
History of stroke, *n* (%)	106 (13.5%)	7 (14.9%)	99 (13.4%)	0.776
Previous medications				
Antiplatelet, *n* (%)	119 (15.2%)	10 (21.3%)	109 (14.8%)	0.229
Statin, *n* (%)	99 (12.6%)	9 (19.1%)	90 (12.2%)	0.165
Clinical data on admission, median (IQR)				
SBP(mmHg)	152.00 (139.00,167.00)	160.00 (141.50, 171.50)	152.00 (139.00, 166.00)	0.133
DBP(mmHg)	84.00 (75.00,94.00)	89.00 (79.00, 97.50)	84.00 (74.00, 93.00)	0.046
NIHSS scores (points)	4.00 (2.00,8.00)	11.00 (4.50, 16.00)	3.00 (2.00, 7.00)	<0.001
OTT(minutes)	145.00 (100.00,190.00)	129.00 (99.00, 191.00)	146.00 (101.00, 190.00)	0.569
RTPA dosage (0.9 mg/kg)	726 (92.6%)	45 (95.7%)	681 (92.4)	0.547
Laboratory tests, median (IQR)				
WBC(*10^9^/L)	7.21 (5.99,8.79)	6.89 (5.93, 9.42)	7.22 (5.99, 8.78)	0.829
Neutrophil(*10^9^/L)	4.38 (3.50,5.77)	4.82 (3.21, 6.14)	4.37 (3.51, 5.75)	0.853
Lymphocyte(*10^9^/L)	1.85 (1.40,2.53)	1.90 (1.14, 2.49)	1.85 (1.43, 2.55)	0.301
RBC(*10^12^/L)	4.62 (4.26,4.95)	4.63 (3.95, 4.91)	4.62 (4.27, 4.95)	0.307
Hemoglobin(g/L)	143.00 (131.00,154.00)	140.00 (124.50, 153.00)	143.00 (132.00, 154.00)	0.390
RDW (%)	12.80 (12.30,13.30)	12.90 (12.60, 13.70)	12.80 (12.30, 13.20)	0.064
Platelet(*10^9^/L)	205.00 (165.75,242.00)	190.00 (160.00, 223.00)	205.00 (166.00, 242.00)	0.149
APTT(s)	34.05 (31.60,36.70)	33.30 (31.45, 35.85)	34.10 (31.70, 36.70)	0.229
Fibrinogen (g/L)	3.21 (2.76,3.72)	3.46 (2.96, 4.20)	3.18 (2.75, 3.69)	0.010
Potassium (mmol/L)	3.81 (3.56,4.05)	3.89 (3.56, 4.27)	3.81 (3.56, 4.04)	0.184
Serum sodium (mmol/L)	139.10 (137.40,141.00)	138.20 (136.00, 140.20)	139.20 (137.40, 141.00)	0.031
Serum calcium (mmol/L)	2.28 (2.23,2.35)	2.27 (2.22, 2.31)	2.29 (2.23, 2.36)	0.116
Blood glucose level (mmol/L)	6.92 (5.98,8.54)	7.15 (6.09, 8.31)	6.92 (5.98, 8.54)	0.732
BUN (mmol/L)	5.80 (4.80,7.20)	6.00 (5.35, 7.75)	5.80 (4.70, 7.20)	0.041
Serum creatinine (μmol/L)	73.00 (61.00,87.00)	77.00 (68.00, 90.50)	72.00 (61.00, 87.00)	0.062
NLR	2.29 (1.55,3.50)	2.74 (1.54, 4.68)	2.28 (1.55, 3.46)	0.351
EIS, *n* (%)	198 (25.3%)	27 (57.4%)	171 (23.2%)	<0.001
Quartiles of Sodium, *n* (%)				0.089
Q1	196 (25.0%)	19 (40.4%)	177 (24.0%)	
Q2	190 (24.2%)	10 (21.3%)	180 (24.4%)	
Q3	187 (23.9%)	8 (17.0%)	179 (24.3%)	
Q4	211 (26.9%)	10 (21.3%)	201 (27.3%)	

sICH, symptomatic intracranial hemorrhage; OTT, onset-to-treatment; NIHSS, National Institutes of Health Stroke Scale; SBP, systolic blood pressure; DBP, diastolic blood pressure; WBC, white blood cell; RBD, red blood cell; RDW, red blood cell distribution; APTT, active patrial thromboplastin; BUN, blood urea nitrogen; NLR, neutrophil-to-lymphocyte ratio; EIS, early infarct signs; RTPA, recombinant tissue plasminogen activator.

### Relationship between serum sodium levels and symptomatic intracranial hemorrhage

**Figure 2:**
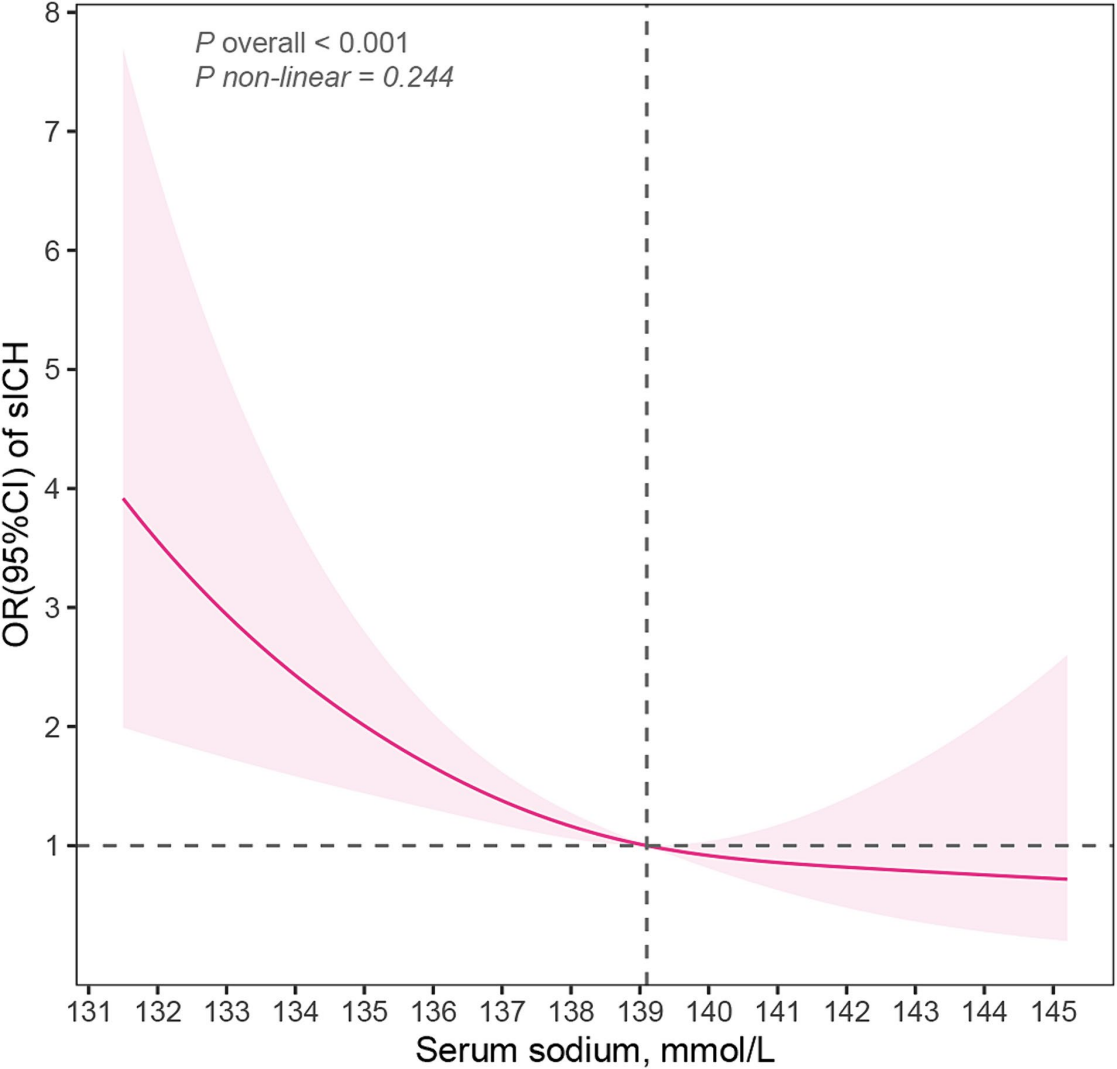
Restricted cubic spline model illustrated the association of serum sodium and sICH (*P* overall <0.001, *P* non-linear = 0.224). The model accounts for confounding factors, including age, sex, atrial fibrillation, NIHSS scores, diastolic blood pressure, early infarct signs, blood urea nitrogen and fibrinogen. sICH, symptomatic intracerebral hemorrhage; NIHSS, National Institutes of Health Stroke Scale; OR, odd ratio; CI, confidence interval.

Table 2 presented the correlation between sICH incidence and serum sodium levels. The analysis revealed a trend that the risk of sICH increased with decreasing serum sodium levels in model3, even after accounting for potential confounding factors such as age, sex, AF, NIHSS scores, diastolic blood pressure, blood urea nitrogen, fibrinogen and EIS (*P* for trend = 0.036). Furthermore, Q3 (serum sodium concentration: 139.1–140.9 mmol/L) exhibited the highest resistance to sICH occurrences compared to Q1 (serum sodium concentration ≤ 137.0 mmol/L) (OR: 0.33, 95% CI: 0.13–0.81) after adjusting all confounding variables. When serum sodium levels were assessed as a continuous variable, the data suggested a 14% reduction in sICH risk for each 1 mmol/L increase in serum sodium concentration in model 3 (OR: 0.85, 95% CI: 0.79–0.93). Figure 2 illustrated the linear dose–response relationship between serum sodium and sICH after adjusting age, sex, AF, NIHSS scores, diastolic blood pressure, blood urea nitrogen, fibrinogen and EIS (*P* overall <0.001, *P* non-linear = 0.244). The investigation revealed a progressive decrease in sICH risk associated with a consistent increase in serum sodium levels. However, when serum sodium levels exceeded 139.1 mmol/L, the relationship between serum sodium and sICH risk became statistically non-significant.

**Table 2:** Association of serum sodium concentrations and symptomatic intracranial hemorrhage.

Serum sodium concentration	Model 1		Model 2		Model 3	
	OR (95% CI)	*p*	OR (95% CI)	*p*	OR (95% CI)	*p*
Per 1 mmol/L	0.88 (0.82,0.95)	<0.001	0.87 (0.81,0.94)	<0.001	0.86 (0.79,0.93)	<0.001
Q1	ref		ref		ref	
Q2	0.52 (0.23,1.14)	0.104	0.44 (0.19,0.99)	0.048	0.43 (0.18,0.99)	0.089
Q3	0.42 (0.18,0.98)	0.044	0.35 (0.15,0.85)	0.020	0.33 (0.13,0.81)	0.032
Q4	0.46 (0.21,1.02)	0.057	0.42 (0.19,0.95)	0.037	0.37 (0.16,0.87)	0.049
*p* for trend		0.039		0.025		0.036

Model 1: Unadjusted.

Model 2: Adjustment for age, sex, atrial fibrillation.

Model 3: Adjustment for Model2, along with NIHSS score, early infarct signs, diastolic blood pressure, blood urea nitrogen and fibrinogen.

### Incremental prognostic value of serum sodium

Table 3 showcased the enhancement in the AUROC for predicting sICH incidence when serum sodium levels was integrated into the traditional models, such as the SEDAN score and GRASPS score (6, 7). The integration of serum sodium levels also led to substantial improvements in continuous NRI, with increases of 0.308 (95%CI: 0.015, 0.602) and 0.368 (95%CI: 0.076, 0.661), respectively (*p* < 0.05).

**Table 3:** Incremental prognostic value of serum sodium levels on symptomatic intracranial hemorrhage.

Model	AUROC (95%CI)	*p* for AUROC comparison	NRI (95%CI)	*p* for NRI
SEDAN score	0.757 (0.687, 0.827)		ref	
SEDAN score+serum sodium	0.800 (0.741, 0.860)	0.089	0.308 (0.015, 0.602)	0.040
GRASPS score	0.728 (0.653, 0.802)		ref	
GRASPS score + serum sodium	0.778 (0.711, 0.845)	0.028	0.368 (0.076, 0.661)	0.014

The SEDAN score included baseline blood sugar, early infarct signs, dense cerebral artery, age and NIHSS score.

The GRASPS score included age, NIHSS score, baseline systolic blood pressure, baseline blood sugar, race and sex.

AUROC, area under the receiver operating characteristic curve; NRI, net reclassification improvement; CI, confidence interval; NIHSS, National Institutes of Health Stroke Scale.

### Subgroup analysis

**Figure 3:**
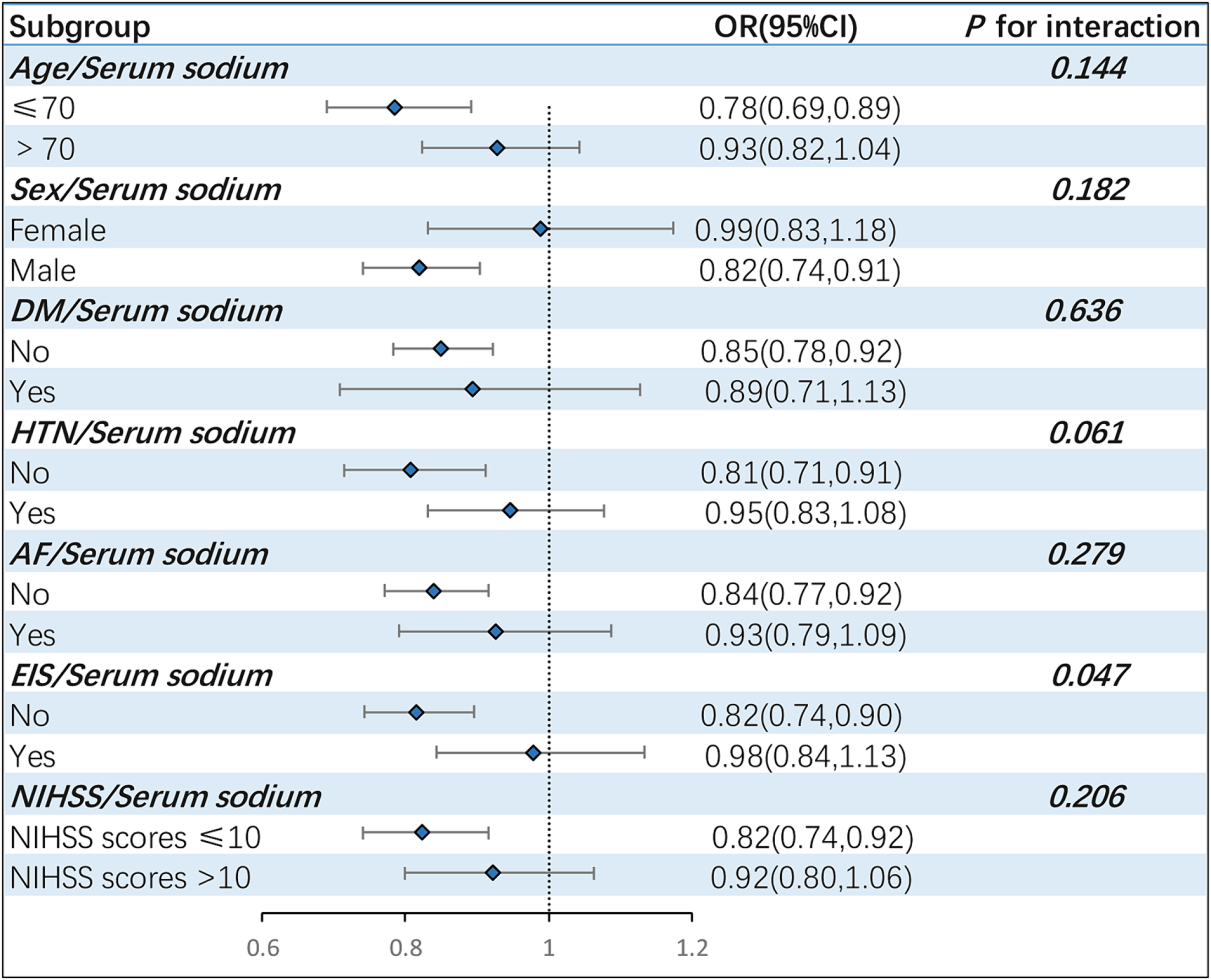
Stratified analysis assessing the interaction effect of serum sodium on sICH incidence across varied subgroups. The forest plot revealed that EIS might exert a moderating influence on the correlation between serum sodium and sICH occurrences (*P* for interaction =0.047). sICH, symptomatic intracranial hemorrhage; DM, diabetes mellitus; HTN, hypertension; AF, atrial fibrillation; EIS, early infarct signs; OR, odd ratio; CI, confidence interval.

Subgroup analyses were conducted, stratifying the patient population by various factors, including age, sex, hypertension, diabetes mellitus, AF, EIS and NIHSS scores (Figure 3). The results indicated that male patients (OR = 0.82, 95% CI: 0.74–0.91) aged 70 or younger (OR = 0.78, 95% CI: 0.69–0.89), without hypertension (OR = 0.81, 95% CI: 0.71–0.91), without diabetes mellitus (OR = 0.85, 95% CI: 0.78–0.92), without atrial fibrillation (OR = 0.84, 95% CI: 0.77–0.92),without early infarct signs (OR = 0.82, 95% CI: 0.74–0.90) and baseline NIHSS scores less than or equal to 10 points (OR = 0.82, 95% CI: 0.74–0.92) were more prone to experience sICH as serum sodium levels decreased. The stratified analysis also suggested that EIS could potentially modulate the relationship between serum sodium and the risk of sICH (*P* for interaction =0.047).

## Discussion

The relationship between hyponatremia and intracranial hemorrhage, particularly subarachnoid hemorrhage, has been extensively studied (25, 26). However, the correlation between serum sodium concentration and post-thrombolysis hemorrhagic transformation in ischemic stroke patients has not been extensively explored. This study shows lower serum sodium concentrations are associated with an increased risk of post-thrombolysis hemorrhagic transformation. The results are consistent with previous research findings suggesting that hyponatremia is associated with an increased risk of post-thrombolysis hemorrhagic transformation in ischemic stroke patients (11).

Moreover, this study further explored the relationship between serum sodium levels and symptomatic intracranial hemorrhage following rtPA intravenous thrombolysis. The incidence of sICH was 6.0%, consistent with prior large-scale studies (27). Regardless of whether serum sodium levels were included in the study as categorical or continuous variables, the incidence of sICH decreased with increasing serum sodium concentration after adjusting for pertinent confounders (*p* < 0.05). However, a recent single-center, retrospective cohort study examining reperfusion therapy for acute ischemic stroke did not find a significant association between hyponatremia and symptomatic intracranial hemorrhage (28). This discrepancy may stem from the inclusion of both thrombolysis and thrombectomy patients in that study, along with an unclear definition of sICH. Additionally, another retrospective, single-center study found no significant correlation between serum sodium levels and sICH in multivariate logistic regression analysis, albeit observing higher serum sodium levels in patients with post-thrombolysis sICH in univariate analysis (29). However, this study focused on ischemic stroke patients with severe white matter lesions. Further studies with high homogeneity are needed to arrive at a conclusive understanding.

To ascertain the optimal serum sodium concentration prior to thrombolysis, this study directly examined the relationship between serum sodium concentration and sICH, rather than hyponatremia and sICH. The lowest risk of sICH after IVT was observed when the serum sodium concentration fell within the range of 139.1–140.9 mmol/L. The RCS suggested a linear relationship between serum sodium levels and the risk of post-IVT sICH (*P* overall <0.001, *P* non-linear = 0.244). However, the dose–response analysis unconverted that the decrease in sICH risk with increasing serum sodium was not statistically significant when the serum sodium level exceeded 139.1 mmol/L. This trend persists even within the normal range of serum sodium levels, as only 11 patients had serum sodium levels above the upper limit of the typical normal range (145 mmol/L) (30). In the logistic regression models, the OR for Q3 (serum sodium concentration: 139.1–140.9 mmol/L) was lower than that for Q4 (serum sodium concentration ≥ 141.0 mmol/L), indirectly supporting the findings of the RCS analysis. Based on the results of RCS and logistic regression, maintaining pre-thrombolysis serum sodium concentration above 139.1 mmol/L can reduce the risk of post-thrombolysis sICH.

Upon establishing the relationship between serum sodium levels and sICH, serum sodium levels were incorporated into classical prediction model for post-thrombolysis sICH. This inclusion led to improvements in both AUROC and NRI compared to the original models. This further validated the strong association between serum sodium and post-IVT sICH. Traditionally, serum sodium was not considered as a potential independent variable in the construction of traditional clinical predictive models or scoring systems (4–7). If serum sodium levels were to be included in the modeling process, would it have a place in the final models or scoring systems?

Subgroup analysis revealed a more pronounced negative correlation between serum sodium concentration and sICH among young male patients without comorbidities. We hypothesize the influence of baseline physiological state or chronic diseases on serum sodium concentration may weaken the relationship between serum sodium concentration and sICH. Older adults are more susceptible to electrolyte disorders compared to younger individuals due to their weaker autonomic regulation (31). Women experience hormonal fluctuations during their menstrual cycle and menopause, which can lead to electrolyte imbalances (32). Diabetic patients often exhibit electrolyte disturbances alongside abnormal glucose metabolism (33). Hypertensive patients may develop hyponatremia due to the use of diuretics and other antihypertensive drugs (33). Electrolyte disturbances can predispose individuals to atrial fibrillation, making it easier to detect atrial fibrillation in hospital (34). An elevated baseline NIHSS score inherently increases the risk of sICH following thrombolysis (5), thereby weakening the association between serum sodium concentration and post-thrombolysis sICH. Furthermore, a significant interaction was identified between EIS and serum sodium concentration in predicting the occurrence of post-thrombolysis sICH (*p* = 0.047). The hyponatremia-induced shift in water from the extracellular to the intracellular compartment can cause cerebral edema (35, 36), intensifying cytotoxic edema in ischemic brain tissue and leading to early detection of brain lesions in cranial NCCT (37, 38). However, lower serum sodium levels were more closely linked to the occurrence of post-thrombolysis sICH in AIS patients without EIS compared to those with EIS. This indirectly suggests that serum sodium may involve alternative mechanisms beyond intensifying cellular edema in the development of sICH. Further basic research or *in vitro* experiments are necessary to explore these mechanisms.

Given the timely availability of serum sodium results before thrombolysis and the potential for medical intervention to regulate serum sodium levels (12, 13), it may be possible to reduce the risk of sICH in thrombolytic patients by managing their serum sodium concentrations. Brain edema is one of the most devastating consequences of ischemic stroke (39) and hyponatremia further exacerbates its occurrence (40). Hypertonic saline and mannitol are recommended for reducing brain edema in patients with spontaneous intracerebral hemorrhage (41). Mannitol, being a diuretic, can lead to intravascular volume loss and hypotension, adversely affecting the perfusion of ischemic brain cells (41). Therefore, hypertonic saline appears advantageous in alleviating cerebral edema in patients with ischemic stroke. Additional research is warranted to determine whether hypertonic saline can effectively mitigate brain edema and subsequently decrease the incidence of sICH in post-thrombolysis ischemic stroke patients.

This study has some limitations. Firstly, this retrospective study was conducted at a single center, potentially introducing biases and limiting the statistical robustness of the findings. Secondly, the study solely observed serum sodium levels at admission and did not track dynamic changes in serum sodium levels. Thirdly, certain variables associated with post-thrombolysis HT, such as serum magnesium levels and neuroimaging biomarkers like cerebral microbleeds (42, 43), were not included in the study, thereby indicating the presence of residual confounding bias. Finally, the study did not elaborate on the relationship between hypernatremia and sICH due to the limited number of cases involving hypernatremia. Further prospective, multicenter studies involving a substantial population are warranted.

## Conclusion

In conclusion, lower serum sodium levels were recognized as an independent risk factor for post-thrombolysis sICH. Maintaining serum sodium concentration above 139.1 mmol/L prior to IVT has the potential to decrease the risk of sICH following IVT. EIS may play a role in modulating the relationship between serum sodium and the risk of sICH. Further randomized control trials are necessary to investigate whether early management of serum sodium levels before IVT could effectively reduce the risk of sICH following IVT.

## Data availability statement

The raw data supporting the conclusions of this article will be made available by the authors, without undue reservation.

## Ethics statement

The studies involving humans were approved by Ethics Committee of Dongyang People’s Hospital. The studies were conducted in accordance with the local legislation and institutional requirements. The ethics committee/institutional review board waived the requirement of written informed consent for participation from the participants or the participants’ legal guardians/next of kin because this is an observational study. In the process of data extraction and analysis, patients’ personal information was concealed to fully protect patient privacy.

## Author contributions

XW: Writing – review & editing, Writing – original draft, Visualization, Validation, Supervision, Software, Project administration, Methodology, Investigation, Formal analysis, Data curation, Conceptualization. ZJ: Writing – review & editing, Writing – original draft, Visualization, Validation, Supervision, Software, Resources, Project administration, Methodology, Investigation, Formal analysis, Data curation, Conceptualization. DX: Writing – review & editing, Validation, Supervision, Project administration. RZ: Writing – review & editing, Validation, Supervision, Methodology. HL: Writing – review & editing, Data curation.
