# Supplementary material for: Pre-thrombolysis serum sodium concentration is associated with post-thrombolysis symptomatic intracranial hemorrhage in ischemic stroke patients
Source: Front Neurol. 2024 May 31;15:1341522. doi: 10.3389/fneur.2024.1341522 (PMC11178046; doi:10.3389/fneur.2024.1341522)
Supplement: Supplementary file 1 [file Table_1.DOCX]

TABLE 1. Baseline patient characteristics categorized by hemorrhagic transformation

| Variables | Total(N=784) | HT(N=149) | Non-HT  (N=635) | *p* |
| --- | --- | --- | --- | --- |
| **Demographic variables** |  |  |  |  |
| Age（years） | 69.50(59.00,78.00) | 74.00(63.00, 79.00) | 69.00(58.00, 78.00) | 0.013 |
| Female, n (%) | 282 (36.0%) | 58 (38.9%) | 224 (35.3%) | 0.403 |
| **Vascular risk factors** |  |  |  |  |
| Smoking, n (%) | 140 (17.9%) | 27 (18.1%) | 113 (17.8%) | 0.926 |
| Drinking, n (%) | 165(21.0%) | 34 (22.8%) | 131 (20.6%) | 0.555 |
| Hypertension, n (%) | 537 (68.5%) | 99 (66.4%) | 438 (69.0%) | 0.549 |
| Diabetes mellitus, n (%) | 141 (18.0%) | 30 (20.1%) | 111 (17.5%) | 0.448 |
| Hyperlipidemia, n (%) | 272(34.7%) | 50 (33.6%) | 222 (35.0%) | 0.746 |
| Ischemic heart disease, n (%) | 116 (14.8%) | 27 (18.1%) | 89 (14.0%) | 0.204 |
| Atrial fibrillation, n (%) | 130 (16.6%) | 35 (23.5%) | 95 (15.0%) | 0.012 |
| History of stroke, n (%) | 106 (13.5%) | 21 (14.1%) | 85 (13.4%) | 0.820 |
| **Previous medications** |  |  |  |  |
| Antiplatelet, n (%) | 119 (15.2%) | 28 (18.8%) | 91 (14.3%) | 0.172 |
| Statin, n (%) | 99（12.6%） | 18 (12.1%) | 81 (12.8%) | 0.823 |
| **Clinical data on admission** |  |  |  |  |
| SBP（mmHg） | 152.00(139.00,167.00) | 156.00(143.00, 168.00) | 152.00(139.00, 166.00) | 0.046 |
| DBP（mmHg） | 84.00(75.00,94.00) | 87.00(77.00, 96.00) | 84.00(74.00, 93.00) | 0.018 |
| NIHSS score | 4.00(2.00,8.00) | 6.00(3.00, 12.00) | 3.00(2.00, 7.00) | <0.001 |
| OTT（minutes） | 145.00(100.00,190.00) | 140.00(101.00, 194.00) | 146.00(100.00, 189.50) | 0.954 |
| RTPA dosage （0.9mg/kg） | 726（92.6%） | 139（93.3%） | 587（92.4） | 0.722 |
| **Laboratory tests** |  |  |  |  |
| WBC(*10^9^/L) | 7.21(5.99,8.79) | 7.18(6.05, 9.34) | 7.22(5.98, 8.69) | 0.427 |
| Neutrophil(*10^9^/L) | 4.38(3.50,5.77) | 4.45(3.50, 6.16) | 4.36(3.50, 5.69) | 0.220 |
| Lymphocyte(*10^9^/L) | 1.85(1.40,2.53) | 1.82(1.25, 2.44) | 1.85(1.44, 2.56) | 0.314 |
| RBC(*10^12^/L) | 4.62(4.26,4.95) | 4.63(4.11, 4.91) | 4.62(4.28, 4.95) | 0.256 |
| Hemoglobin(g/L) | 143.00(131.00,154.00) | 144.00(127.00, 153.00) | 143.00(132.00, 154.00) | 0.461 |
| RDW (%) | 12.80(12.30,13.30) | 12.80(12.30, 13.50) | 12.80(12.40, 13.20) | 0.406 |
| Platelet(*10^9^/L) | 205.00(165.75,242.00) | 205.00(165.00, 250.00) | 205.00(166.00, 241.00) | 0.907 |
| APTT(s) | 34.05(31.60,36.70) | 33.40(31.40, 36.10) | 34.20(31.80, 36.80) | 0.050 |
| Fibrinogen (g/L) | 3.21(2.76,3.72) | 3.37(2.89, 3.81) | 3.15(2.73, 3.69) | 0.008 |
| Potassium (mmol/L) | 3.81(3.56,4.05) | 3.82(3.56, 4.16) | 3.81(3.57, 4.04) | 0.314 |
| Serum sodium (mmol/L) | 139.10(137.40,141.00) | 138.50(136.80, 140.20) | 139.50(137.70, 141.00) | 0.004 |
| Serum calcium (mmol/L) | 2.28(2.23,2.35) | 2.28(2.23, 2.33) | 2.29(2.23, 2.35) | 0.214 |
| Blood glucose level (mmol/L) | 6.92(5.98,8.54) | 7.00(5.97, 8.53) | 6.89(5.98, 8.54) | 0.731 |
| BUN (mmol/L) | 5.80(4.80,7.20) | 5.90(4.90, 7.40) | 5.80(4.70, 7.20) | 0.481 |
| Serum creatinine (μmol/L) | 73.00(61.00,87.00) | 70.00(59.00, 88.00) | 73.00(62.00, 87.00) | 0.786 |
| NLR | 2.29(1.55,3.50) | 2.49(1.56, 3.89) | 2.25(1.54, 3.41) | 0.165 |
| EIS, n (%) | 198 (25.3%) | 74 (49.7%) | 124 (19.5%) | <0.001 |
| Quartiles of Sodium, n (%) |  |  |  | 0.001 |
| Q1 | 196 (25.0%) | 53 (35.6%) | 143 (22.5%) |  |
| Q2 | 190 (24.2%) | 40 (26.8%) | 150 (23.6%) |  |
| Q3 | 187 (23.9%) | 23 (15.4%) | 164 (25.8%) |  |
| Q4 | 211 (26.9%) | 33(22.1%) | 178(28.0%) |  |

**Abbreviations:** HT, hemorrhagic transformation; OTT, onset-to-treatment; NIHSS, National Institutes of Health Stroke Scale; SBP, systolic blood pressure; DBP, diastolic blood pressure; WBC, white blood cell; RBD, red blood cell; RDW, red blood cell distribution; APTT, active patrial thromboplastin; BUN, blood urea nitrogen; NLR, neutrophil-to-lymphocyte ratio; EIS, early infarct signs; RTPA, recombinant tissue plasminogen activator.
