# Supplementary material for: Pre-thrombolysis serum sodium concentration is associated with post-thrombolysis symptomatic intracranial hemorrhage in ischemic stroke patients
Source: Front Neurol. 2024 May 31;15:1341522. doi: 10.3389/fneur.2024.1341522 (PMC11178046; doi:10.3389/fneur.2024.1341522)
Supplement: Supplementary file 2 [file Table_2.DOCX]

**Table 2.** Association of serum sodium concentrations and hemorrhagic transformation

| Serum sodium  concentration | Model 1  OR (95% CI) | *P* | Model 2  OR (95% CI) | *P* | Model 3  OR (95% CI) | *P* |
| --- | --- | --- | --- | --- | --- | --- |
| Per1mmol/L | 0.93(0.88,0.98) | 0.005 | 0.92(0.87,0.97) | 0.003 | 0.91(0.86,0.96) | 0.001 |
| Q1 | ref |  | ref |  | ref |  |
| Q2 | 0.72(0.45,1.15) | 0.170 | 0.68(0.42,1.09) | 0.113 | 0.71(0.43,1.18) | 0.191 |
| Q3 | 0.38(0.22,0.65) | <0.001 | 0.35(0.20,0.60) | <0.001 | 0.32(0.18,0.57) | <0.001 |
| Q4 | 0.50(0.31,0.81) | 0.005 | 0.47(0.29,0.77) | 0.003 | 0.43(0.25,0.73) | 0.002 |
| *P for trend* |  | 0.001 |  | <0.001 |  | <0.001 |

Model1：Unadjusted model

Model2：Adjustment for age, sex, atrial fibrillation

Model3：Adjustment for Model2, along with NIHSS score, early infarct signs, systolic blood pressure, diastolic blood pressure and fibrinogen.
